# Synthesis and tumour cell uptake studies of gadolinium(III)–phosphonium complexes

**DOI:** 10.1038/s41598-020-79893-9

**Published:** 2021-01-12

**Authors:** Andrew J. Hall, Amy G. Robertson, Leila R. Hill, Louis M. Rendina

**Affiliations:** 1 School of Chemistry, The University of Sydney, Sydney, NSW 2006 Australia; 2 The University of Sydney Nano Institute, Sydney, NSW 2006 Australia

**Keywords:** Coordination chemistry, Inorganic chemistry, Medicinal chemistry

## Abstract

The synthesis of a new series of Gd(III)-arylphosphonium complexes is described and the solution stability of selected compounds is reported. Their lipophilicity and uptake in human glial (SVG p12) and human glioblastoma multiforme (T98G) cell lines are presented. The in vitro cytotoxicity of all complexes was determined to be low at therapeutically-relevant concentrations. Selected Gd(III) complexes are potential candidates for further investigation as theranostic agents.

## Introduction

Phosphonium-based delocalized lipophilic cations (DLCs) demonstrate selective accumulation in mitochondria with a high membrane potential (Δ*Ψ*_m_), an attribute associated with the mitochondria of aggressive tumours, and have consequently shown promise as cancer targeting vectors^1–3^. When coupled with relevant lanthanoid chelators these phosphonium DLCs are able to selectively shuttle medically-relevant metal ions into the mitochondria of these cancer cells independent of endogenous transporters^4–6^. Initial investigations of these ligands have demonstrated their promise as candidates for the development of Gd(III)-based theranostics^7–9^.

Gd(III) has a number of unique properties that support its use in theranostic agents. Owing to the highly paramagnetic nature of Gd(III), this ion is a key component of contrast agents for magnetic resonance imaging (MRI)^10–12^. The naturally-occurring (15.7% abundance) and non-radioactive ^157^Gd isotope is also an excellent candidate for neutron capture therapy (NCT) as it possesses the largest neutron capture cross-section of any stable isotope. Upon thermal neutron capture by the ^157^Gd nucleus, the resulting excited ^157^Gd* nucleus undergoes a nuclear re-arrangement and subsequent production of a prompt *γ*-photon, internal-conversion electrons, and *ca*. 5 high-energy Auger and Coster-Kronig (ACK) electrons per neutron capture event. The ACK electrons appear to be the key therapeutically-relevant particles^10,13,14^. The potential use of ^157^Gd in the related neutron capture enhanced particle therapy (NCEPT) has recently been reported^15^. Due to its high atomic number (*Z*), Gd also shows great potential in photon activation therapy (PAT), more specifically synchrotron stereotactic radiotherapy (SSR), given its achievable *K*-edge of 50.2 keV, resulting in an average of *ca*. 8 ACK electrons per photon activation event^14,16^. Localisation within critical subcellular components is required for the high efficacy of Gd therapeutics that produce ACK electrons due to their high linear energy transfer (LET) nature, depositing their energy over very small path lengths (*ca.* 12.5 nm)^14,17^.

Recent studies demonstrating Gd deposition in the brain tissue of individuals who have received consecutive administrations of Gd-based MRI contrast agents have concerned radiologists from the US NIH, and these concerns have been recently highlighted by the US FDA^18–25^. Radbruch et al*.* have proposed that differences in Gd deposition are attributable to the thermodynamic stability of the Gd agents administered to the patient, hence there is a critical need to ensure good stability of any Gd agents developed with a clinical application^19,20^.

1,4,7,10-Tetraazacyclododecane (cyclen) is a versatile macrocycle and its *N*-acetic acid derivatives have demonstrated some of the highest stability constants with lanthanoids, facilitating Gd(III) complexation in the centre of a distorted square antiprism^26,27^. 1,4,7,10-Tetraazacyclododecane-1,4,7,10-tetraacetatogadolinium(III) (Gd-DOTA) complexes possesses the highest thermodynamic stability of these derivatives with a log*K* of *ca*. 25.3 resulting from their octadentate coordination^26,28^. Previous work investigating Gd(III)-phosphonium complexes has utilised 1,4,7,10-tetraazacyclododecane-1,4,7-tris(*tert*-butyl acetate) (DO3A) as the remaining unfunctionalised secondary amine within the cyclen structure allows for facile functionalisation of the complex, but it can result in a lower stability with log*K* values between 21.0 and 23.8^26,28^. Herein we report the synthesis and a limited stability and SAR study of a novel series of Gd(III)-DOTA-phosphonium complexes. The uptake of these complexes in human glial (SVG p12) and human glioblastoma multiforme (T98G) cell lines is also presented.

## Results and discussion

Novel Gd(III) complexes (**1**–**5**) were synthesised utilising phosphonium mitochondrial-targeting vectors based upon related prototype Gd(III) complexes that we have reported previously^8,9^. Notably, the inclusion of an additional *O*-donor group attached to the DO3A macrocycle helps facilitate octadentate coordination of the Gd(III) ion and thus should provide enhanced solution stability for the complexes. In addition, a new series of complexes (**6**–**10**) featuring an alkyl linking group of varying chain length was synthesised with the primary aim of identifying any relationship between the nature of the bridging ligand and the degree of cellular uptake and tumour-cell selectivity.

### Synthesis

**Figure 1:**
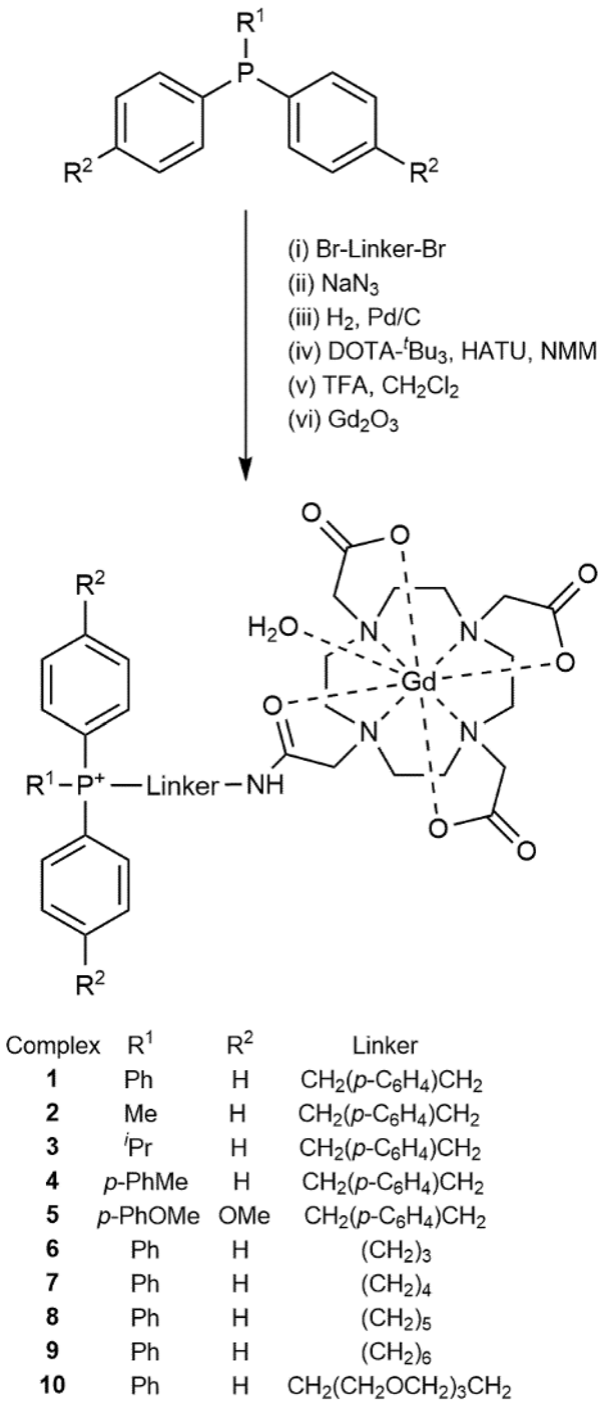
General synthetic scheme for the preparation of complexes **1**–**10** incorporating a DOTA chelating group.

Complexes **1**–**10** were prepared by means of a multistep synthesis that led to the formation of a key amide bond (Fig. 1) followed by complexation of the Gd(III) ion. Briefly, arylphosphines underwent a nucleophilic substitution reaction with one equivalent of a dibrominated linear alkyl, TEG (triethylene glycol) or xylyl group to afford the corresponding mono-substituted phosphonium salts. In these syntheses, it was necessary to exchange the bromine functionality of the phosphonium salt intermediate for an azide group by means of a nucleophilic substitution reaction, followed by hydrogenation to afford the corresponding primary amine. Amide bond formation between the amine precursors and the carboxylic acid functionality of 1,4,7,10-tetraazacyclododecane-1,4,7-tris(*tert*-butylacetate)-10-acetic acid was performed using peptide coupling reagents (hexafluorophosphate azabenzotriazole tetramethyl uronium (HATU) and *N*-methylmorpholine (NMM)) to afford the protected phosphonium ligands. Acid deprotection of the *tert*-butyl groups with trifluoroacetic acid (TFA) and subsequent purification by means of reverse-phase HPLC afforded the free macrocyclic ligands. Complexation of the Gd^3+^ ion was performed by employing a suspension of Gd_2_O_3_ in H_2_O to give the target Gd(III) complexes **1**–**10** in high yield and purity. The purity of each complex (> 95%) was confirmed by means of analytical reverse-phase HPLC, and their identities were confirmed by means of ESI-FTICR-MS or MALDI-TOF–MS.

### Solution stability of Gd(III) complexes

The Gd(III) complexes were found to be highly soluble in water, with selected examples as follows: **1**, 80 mg mL^−1^; **2**, 70 mg mL^−1^; **4**, 40 mg mL^−1^; **6**, 60 mg mL^−1^ and **9**, 250 mg mL^−1^. Complexes **1**–**10** also demonstrated excellent stability in aqueous media at pH 7.4 following incubation at 37 °C for prolonged periods (days), as confirmed by MALDI-TOF–MS. In addition, a 20 mM aqueous sample of complex **6** was analysed by HPLC and found to be > 95% intact after 3 years in solution, indicating excellent long-term stability. During this time, the pH of the solution decreased from 7.4 to *ca.* 5.5. However, decreased stability of complexes at lower pH values for the related DO3A-phosphonium complexes is well-documented^29^. This poor stability in acidic conditions is largely attributed to protonation of the macrocyclic ring nitrogen atoms which decreases the affinity of the ligand for Gd^3+^ ions^29^. Whilst the proposed biological application of these Gd(III) complexes would be dominated by physiological pH (7.4) and the slightly alkaline environment of the mitochondria (~ pH 8), one must also consider the most acidic cellular regions these complexes might encounter^30^. Lysosomes, for example, are acidic organelles that contain a battery of degradative enzymes. They are found in all cells, estimated to make up approximately 1% of the total cellular volume, and maintain a pH ~ 4.8 by proton pumps^30,31^. Whilst the Gd(III) complexes are unlikely to be actively taken up by the lysosomes, it is possible that some molecules may be passively exposed to the hostile acidic environment these organelles foster where they are more likely to undergo demetallation or transmetallation processes that might lead to the release of free Gd^3+^ ions.

**Figure 2:**
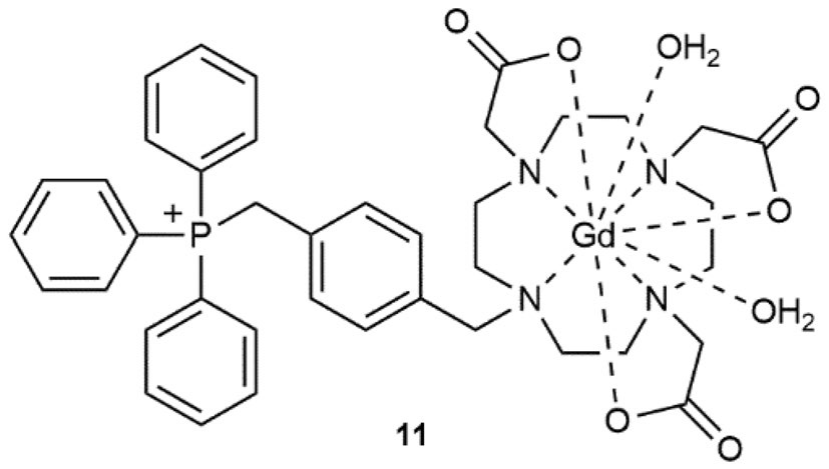
Structure of the previously-reported Gd(III)-DO3A complex **11**.

In this work, the relative stability of complexes **6** and **7** were compared to the previously-reported archetypal Gd(III)-DO3A triphenylphosphonium complex (**11**, shown in Fig. 2) at acidic pH by means of a competition (challenge) assay using the lanthanoid(III) ion indicator xylenol orange^32^. These assays were performed under mildly acidic conditions to ensure the phenolic hydroxyl was protonated prior to chelation^32^. A summary of the results for assays at pH 5.0 and 5.8 are presented in Table 1.

**Table 1:** Competitive stability of complexes **1** and **11**.

pH	Complex	Gradient^a^	*r*^2^ ^b^	% Gd^3+^ retention^c^
5.0	GdCl_3_	3.83 × 10^−2^	0.998	0
5.0	**1**	1.67 × 10^−2^	0.971	56.5 ± 3.4
5.0	**11**	3.34 × 10^−2^	0.995	12.8 ± 0.4
5.8	GdCl_3_	3.57 × 10^−2^	0.993	0
5.8	**1**	1.82 × 10^−2^	0.978	49.0 ± 2.8
5.8	**11**	2.94 × 10^−2^	0.998	17.6 ± 0.6

^a^Slope of the line of best fit resulting from a plot of the average data using 10 different concentrations (2.0–250 μM).

^b^Regression analysis of the line of best fit.

^c^Percentage of Gd^3+^ retained by the phosphonium complex over a period of 24 h, as determined by the shift in absorbance from 433 to 573 nm, averaged across all data points.

Linear regression analysis of the data found a significant difference (*p* < 0.01) between the control group (i.e. the Gd^3+^ salt GdCl_3_) and the Gd(III)-DO3A analogue (**11**), as well as between **1** and **11** and at pH 5.0 and 5.8. These results demonstrate that under mild acidic conditions that are typically found in lysosomes, in the presence of the competitive chelator (xylenol orange), the stability of the Gd(III)-DOTA complex **1** is significantly greater (*ca.* 3 to 4 times) than that of the previously-reported DO3A analogue **11**. For those experiments that were run at lower pH values (i.e. pH < 4.0), the readings did not exceed the baseline absorbance values (573 nm) at any meaningful levels, consistent with complex demetallation.

### MRI relaxivity of representative complex **1**

Relaxivity studies were performed on the representative parent complex **1** and calculations showed that *r*_1_ = 7.0 ± 1.1 mM^−1^ s^−1^ (9.4 T, 298 K) which is somewhat higher than the value reported for the Gd-DO3A analogue **11** (5.1 mM^−1^ s^−1^)^8^. Furthermore, the relaxivity value of **1** lies at the higher end of clinical Gd MRI contrast agents such as Magnevist, probably related to the higher MW of **1** compared to that of clinical contrast agents. The complete relaxivity data are presented in Table S6 and Figure S1 (Supplementary Information).

### Determination of Gd(III) complex lipophilicity

Lipophilicity is a vital parameter to consider regarding DLCs as it underpins their membrane permeability, and also strongly influences absorption, distribution, metabolism and excretion of the Gd(III)-phophonium complex within the body^33,34^. Hence, it is important to consider and analyse the effect that the lipophilicity of these complexes has on their efficacy to assist in future Gd theranostic design. Log*P* (where *P* is the partition coefficient) is the most common measurement of lipophilicity and it was determined for **1**–**10** by means of a reverse-phase HPLC method involving a suite of standard reference compounds. A summary of the results is shown in Table 2.

**Table 2:** Experimentally-determined log*P* values for complexes **1**–**11** at pH 7.4

Complex	Log*P* ^a^
**1**	1.12 ± 0.20
**2**	1.19 ± 0.19
**3**	1.23 ± 0.18
**4**	1.41 ± 0.18
**5**	1.63 ± 0.17
**6**	1.39 ± 0.25
**7**	1.35 ± 0.24
**8**	1.38 ± 0.26
**9**	1.39 ± 0.24
**10**	1.37 ± 0.12
**11**	1.24 ± 0.02^b^

^a^Mean ± SEM (N = 3).

^b^Taken from Ref.^8^.

All of the Gd(III) complexes prepared in this work were shown to be reasonably lipophilic, with log*P* values ranging from 1.12 to 1.63. These values are consistent with those previously reported for a range of related DO3A-phosphonium complexes and lie within the pharmacologically optimal value of 1–3, as outlined by Waring et al.^7,8,33^. Furthermore, these log*P* values lie within the predictive model on the optimised uptake of lipophilic cations into the mitochondria reported by Trapp and Horobin^35^. This model indicates the optimal lipophilicity for DLCs lie above log*P* =  − 2, precluding any kinetically-limited uptake due to the high energy of desolvation, and also fall below log*P* = 2 in order to avoid non-specific lipid binding^35^.

### In vitro cytotoxicity studies

In vitro cytotoxicity was determined for each of the new complexes by means of standard 3-(4,5-dimethylthiazol-2-yl)-2,5-diphenyltetrazolium bromide (MTT) colourimetric assays over a 62.5 μM–2 mM range. A summary of the results in Table 3 reports the half-maximal inhibitory concentration (IC_50_) of each of the assessed complexes for the human glial (SVG p12) and human glioblastoma multiforme (T98G) cell lines. Ideally, Gd theranostic agents should demonstrate relatively low cytotoxicity to ensure any uptake into healthy tissue causes only minimal damage, as the intended cytotoxic event for Gd-based binary agents results from either the thermal neutron (NCT/NCEPT) or X-ray photon (PAT) irradiation.

**Table 3:** IC_50_ values for complexes **1**–**11** in SVG p12 and T98G cell lines (*N* = 9).

Complex	IC_50_ (mM)	
	SVG p12	T98G
**1**	2.09 ± 0.15 ^a^	1.72 ± 0.08
**2**	2.10 ± 0.12 ^a^	1.31 ± 0.04
**3**	1.76 ± 0.07	1.17 ± 0.05
**4**	1.19 ± 0.04	1.19 ± 0.04
**5**	1.63 ± 0.07	1.32 ± 0.04
**6**	2.06 ± 0.08 ^a^	1.81 ± 0.06
**7**	1.58 ± 0.04	1.20 ± 0.06
**8**	1.87 ± 0.06	1.78 ± 0.04
**9**	1.98 ± 0.05	1.39 ± 0.04
**10**	> 2.00^b^	> 2.00^b^
**11**	2.13 ± 0.09^a^	2.55 ± 0.33^c^

^a^IC_50_ values were determined by means of the GraphPad Prism 7 non-linear regression analysis tool.

^b^Value too large to accurately predict using available data between 62.5 μM and 2 mM.

^c^Value reported previously (N = 4) in Ref.^8^.

The IC_50_ values for complexes **1**–**10** were found to lie in the low mM range, in agreement with those previously-reported for related DO3A complexes^7–9^. A one-tailed Mann–Whitney U test demonstrated that these complexes were slightly more cytotoxic toward the T98G cells over their healthy SVG p12 cell line, albeit the overall toxicity of the complexes was only marginal (mM).

In order to evaluate the relative cytotoxicities of complexes **1**–**10** with a known (albeit no longer clinically-used) Gd theranostic, Motexafin-Gd (MGd), was chosen. In vitro cell experiments involving MGd were reported to result in 50% cell arrest of the HF1 tumour cell line at concentrations of less than 100 μM, and the Gd agent exhibited significant cytotoxicity at concentrations of *ca*. 50 μM (in the absence of any X-ray irradiation)^36,37^. Evidently, complexes **1**–**10** appear to exhibit far lower in vitro cytotoxicities than the clinical agent MGd.

### In vitro cellular uptake of Gd

**Figure 3:**
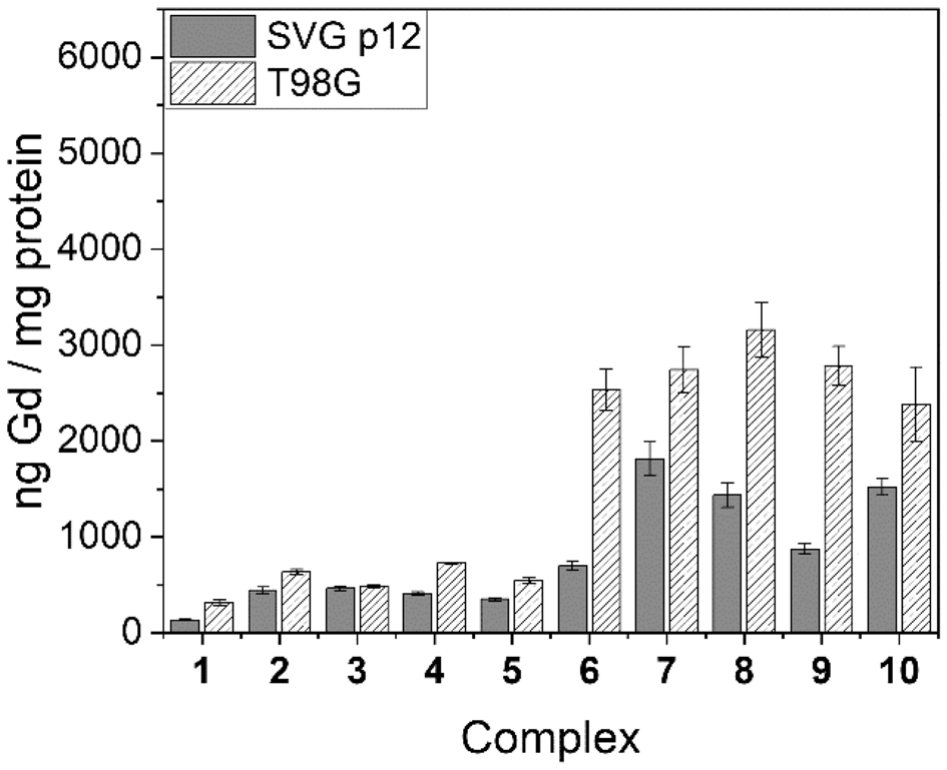
Cell uptake values for complexes **1**–**10** in SVG p12 and T98G cell lines at 125 μM.

**Figure 4:**
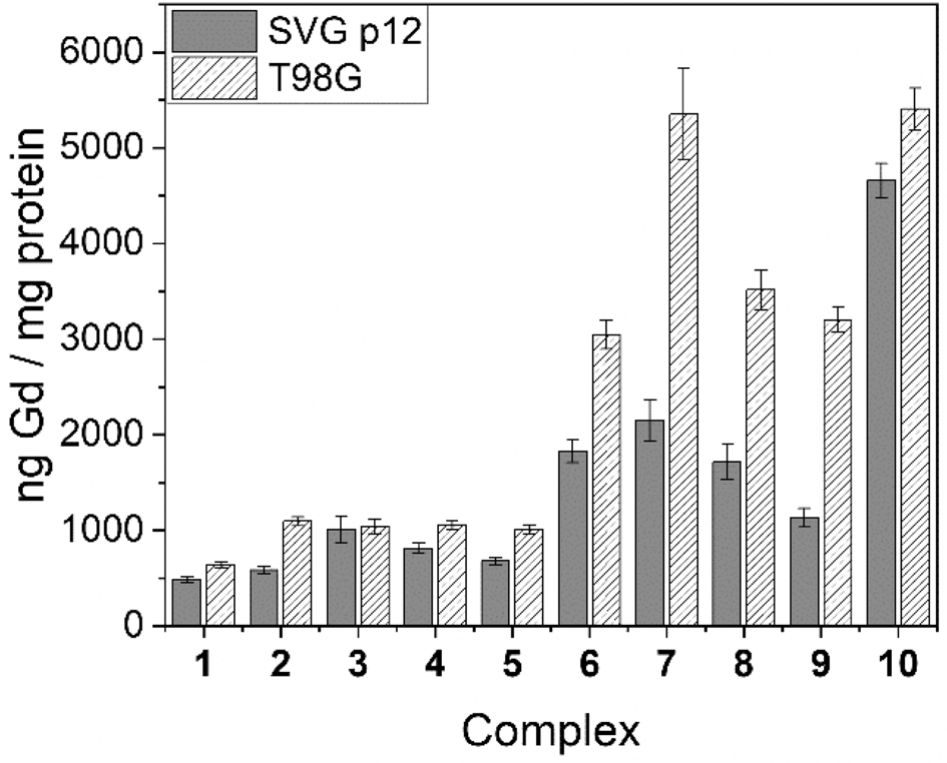
Cell uptake values for complexes **1**–**10** in SVG p12 and T98G cell lines at 250 μM.

**Figure 5:**
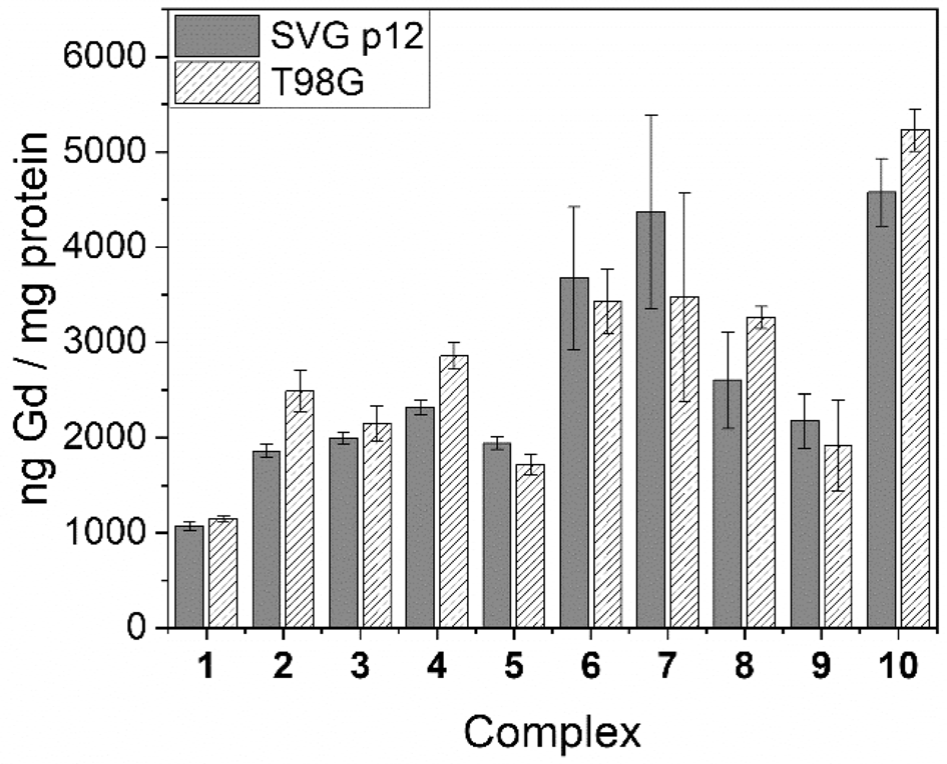
Cell uptake values for complexes **1**–**10** in SVG p12 and T98G cell lines at 500 μM.

As the therapeutic application of Gd(III) complexes relies upon maximising Gd accumulation within the mitochondria of the target tumour cells, comprehensive in vitro cellular uptake studies were performed on complexes **1**–**10** using both SVG p12 and T98G cell lines at four different concentrations (complexes **1**–**5**: 125 μM, 250 μM, 500 μM, 1 mM; complexes **6**–**10**: 62.5 μM, 125 μM, 250 μM, 500 μM). The harvested cells were analysed for accumulated Gd by means of ICP-MS and these values were normalised to protein content, as determined by means of a modified Lowry protein assay. Representative cell uptake results are presented in Figs. 3, 4, 5 (and Supplementary Tables S1–S5) and are expressed as ng Gd/mg protein.

Figures 3, 4, 5 demonstrate that uptake of complexes **1**–**10** occurs in both tumour and healthy cell lines. Table 4 highlights the tumour-to-normal cell uptake ratios. Statistical analysis of the data was performed by means of a two-way ANOVA, demonstrating significantly greater uptake of Gd in the T98G glioma cell line compared with the SVG p12 glial cell line for complexes **2** and **6**–**9** but no statistically significant difference was found between the two cell lines for complexes **1**, **3**–**5** and **10**. Indeed, for complexes **1**–**3**, the tumour cell uptake and selectivity results are not as significant as our previous research reporting related DO3A complexes whereby a reduced delocalisation at the phosphonium centre in moving from, for example, R^1^ = Ph (**1**) to Me (**2**) was strongly correlated with a reduced tumour-cell selectivity and significantly increased Gd uptake^9^. Furthermore, statistical analysis demonstrated that at the lowest treatment dose for each Gd(III) complex (with the exception of complex **3**), a significantly higher Gd uptake in the T98G tumour cell line over the SVG p12 cell line was found. This was also true at the second-lowest treatment dose for each complex, with the exception of the triethylene glycol (TEG) complex **10** which was found to exhibit high tumour cell selectivity (2.8) but only at the lowest treatment dose (62.5 μM). However, the propyl-bridged complex **6** exhibited the highest tumour cell selectivity (3.7) of all complexes studied in this work (at 125 μM). None of the complexes examined in this work demonstrated any significant selectivity for the T89G cell line at the two highest treatment doses (500 μM or 1 mM). The tendency for lower concentration treatment doses resulting in higher Gd uptake into the tumour cells over the healthy cells is consistent with the tumour cell’s elevated Δ*Ψ*_m_ which is responsible for tumour-selective accumulation up to a point whereby high levels of Gd complex accumulation within mitochondria leads to depolarisation, after which passive Gd influx may become the dominant mechanism of mitochondrial accumulation^2,38,39^. This hypothesis is further supported by the tendency that the highest concentration treatment resulted in no significant difference in Gd uptake between the two different cell lines.

**Table 4:** Tumour-to-normal (T:N) cell ratios for complexes **1**–**10**.

Complex	Concentration (μM)	T:N	Complex	Concentration (μM)	T:N
**1**	62.5	–	**6**	62.5	1.7
	125	2.4		125	3.7
	250	1.3		250	1.7
	500	1.1		500	0.9
	1000	1.3		1000	–
**2**	62.5	–	**7**	62.5	1.7
	125	1.4		125	1.5
	250	1.9		250	2.5
	500	1.3		500	0.8
	1000	1.3		1000	–
**3**	62.5	–	**8**	62.5	2.3
	125	1.1		125	2.2
	250	1.0		250	2.1
	500	1.1		500	1.3
	1000	1.6		1000	–
**4**	62.5	–	**9**	62.5	2.2
	125	1.8		125	3.2
	250	1.3		250	2.8
	500	1.2		500	0.9
	1000	1.1		1000	–
**5**	62.5	–	**10**	62.5	2.8
	125	1.6		125	1.6
	250	1.5		250	1.2
	500	0.9		500	1.1
	1000	1.1		1000	–

The electron-rich P-aryl rings in complexes **4** and **5** led to a somewhat increased cell uptake compared to the parent **1** at lower treatment doses, but no significant improvement in tumour-cell selectivity was observed. Furthermore, replacement of the archetypal xylyl linker in **1** with various alkyl and TEG linkers (**6**–**10**) resulted in a significant increase in Gd uptake and tumour cell selectivity at lower treatment doses. In general, no statistically significant trend was established for complexes **6**–**10**, and Gd uptake did not appear to be related to the relative distance between the phosphonium and Gd centres, although a very high Gd uptake was observed for **7** (5349.0 ± 478.1 ng Gd/mg protein), containing the *n-*butyl linker, at one of the assessed doses (250 μM). The incorporation of a hydrophilic TEG linker in complex **10**, however, did lead to an increased Gd uptake at higher concentrations (250 and 500 μM) when compared to the more lipophilic alkyl linkers found in **6**–**9**. However, the higher Gd uptake in **10** is countered by the reduced tumour cell selectivity when compared to most other Gd(III) complexes assessed in this study, except at the lowest treatment dose (62.5 μM).

## Conclusions

Complexes **1**–**10** were synthesised in good to high yields and in high purity. Stability assays comparing the DOTA-based complex **1** to the previously-reported, archetypal DO3A-phosphonium complex **11** at mildly acidic pH conditions demonstrated a significant increase in the stability of **1**, and these findings are consistent with those previously reported for Gd(III)-DOTA complexes by Caravan et al.^26^. Complexes **1**–**10** were all found to have low (mM) cytotoxicity at therapeutically-relevant concentrations, with significant in vitro tumour cell uptake and reasonable tumour cell selectivity at lower concentrations. The reduced tumour cell selectivity of the complexes at higher concentrations is consistent with previous findings which proposes a different mechanism for uptake beyond a certain threshold, although it may also be indicative of Δ*Ψ*_m_ depolarisation^9^.

No significant trends were observed in cell uptake when various phosphonium targeting vectors were used (**1**–**5**), in direct contrast to previous studies involving Gd(III)-DO3A-phosphonium complexes where, for example, the DO3A analogue of **2** was found to have very high T98G cell uptake compared to the parent triphenylphosphonium complex but with a much lower tumour cell selectivity^7–9^. Similarly, a related trend was also observed for the alkyl and TEG linker complexes **6**–**10** which were found to have reduced tumour cell selectivities but showed significantly higher Gd uptake than the *para*-xylyl linker complexes **1**–**5** in both cell lines but even more so in the T98G tumour line. Indeed, the *para*-xylyl DOTA complexes **1**–**5** were not found to differ significantly from their previously-reported DO3A counterparts in terms of cytotoxicity and tumour cell uptake^7–9^, although, in general, tumour cell selectivity was found to be reduced for the DOTA family of complexes, a factor that might be related to a decreased lipophilicity and diminished ability to traverse both the cell and mitochondrial membranes owing to the presence of the amide linkage. However, the log*P* values in Table 2 indicate that the lipophilicities of the DO3A and DOTA complexes are very similar and, indeed, the log*P* difference between the archetypal complexes **1** and **11** is only marginal at best. In vivo uptake and biodistribution studies with selected Gd(III) complexes are currently underway and the results of this work will be reported in due course.

## Methods

All precursor chemicals were commercially available. For experiments requiring H_2_O, ultrapure H_2_O was collected from a Milli-Q water purification system. Anhydrous MeCN, DMF, and PhMe were obtained using a Puresolv system. Et_2_O was dried over sodium wire, distilled and stored under N_2_ over microsieves. MeOH and EtOH were stored under N_2_ over 4 Å sieves. All other solvents were used without further purification. Reactions requiring an inert atmosphere were performed under dry N_2_ and employed conventional Schlenk techniques^40^.

### Characterisation

All ^1^H, ^13^C{^1^H}, and ^31^P{^1^H} NMR spectra were recorded at 300 K on a Bruker Avance 300 (5 mm QNP probe, ^1^H at 300 MHz, ^13^C at 75 MHz, and ^31^P at 121 MHz), a Bruker Avance III 400 (5 mm BBFO probe, ^1^H at 400 MHz, ^13^C at 100 MHz, and ^31^P at 162 MHz), or a Bruker Avance III 500 (5 mm BBFO probe, ^1^H at 500 MHz, ^13^C at 125 MHz, and ^31^P at 202 MHz). All NMR signals (δ) are reported in ppm. ^1^H and ^13^C NMR spectra were referenced according to their solvent residual peaks. ^13^C{^1^H} NMR spectra in D_2_O were referenced according to an internal standard of DMSO-*d*_6_ (39.39 ppm). ^31^P{^1^H} NMR spectra were referenced to external 1% TMS in CDCl_3_ using the unified reference scale^41^. Coupling constants are reported in Hz. Peak multiplicities have been abbreviated as s (singlet), d (doublet), t (triplet), q (quartet), qi (quintet), br (broad), and m (multiplet – unassignable multiplicity). Proton and carbon positions within aryl rings have been abbreviated *i*- (1, 1), *o*- (1, 2), *m*- (1, 3), and *p*- (1, 4). The xylyl linker has been abbreviated to ‘xy’ for clarity’s sake when assigning proton and carbon nuclei belonging to this linker group.

FT-IR spectra were run on a benchtop Bruker ALPHA FTIR spectrometer. All 96 and 384 well plates were read using a BMG LABTECH POLARstar Omega UV spectrometer. Low resolution ESI–MS were recorded on a Bruker amaZon SL mass spectrometer. MALDI-MS were recorded on a Bruker autoflex speed LRF MALDI-TOF mass spectrometer. High resolution ESI-FT-ICR-MS data were recorded on a Bruker Apex Qe 7 T FTICR mass spectrometer. Quantitative MS was recorded on a Perkin Elmer NexION 350X ICP-MS with a quadrupole analyser.

Stock solutions of TA30 solvent (30:70 *v/v* MeCN : 0.1% TFA in water) saturated with α-cyano-4-hydroxycinnamic acid (4CCA) matrix were prepared. The samples were prepared at a concentration of 2 mg mL^−1^ in TA30. MALDI-TOF MS samples were prepared by combining 20 μL of matrix solution with 4 μL of sample solution. 0.5 μL of this sample was deposited on a Ground Steel MALDI target plate and allowed to air dry. Stock solutions of *trans*-2-[3-(4-*tert*-butylphenyl)-2methyl-2-propenylidene]malononitrile (DCTB) (10 mg mL^−1^) in 50:50 DCM:MeCN were prepared. The samples were prepared at a concentration of 2 mg mL^−1^ in DCM. MALDI-TOF MS samples were prepared by combining 20 μL of matrix solution with 4 μL of sample solution. 0.3 μL of this sample was deposited on a Ground Steel MALDI target plate and allowed to air dry.

### HPLC methods

All HPLC methods were performed using a variety of Waters HPLC systems equipped with Waters 2695 separation modules and tuneable absorbance UV/vis detectors (λ = 400–230 nm). Preparative separations utilised a Waters Sunfire C18 preparative column (100 Å, 5 μm, 19 × 150 mm) and a flow rate of 7 mL min^−1^. The mobile phase consisted of two solutions: H_2_O with 0.1% TFA and MeCN with 0.1% TFA, run in a gradient from 100% H_2_O to 100% MeCN usually over 45 min. Semi-preparative separations were selected when experiments yielded side products that eluted without considerable baseline separation in preparative methods, and utilised a Waters Sunfire C18 semi-preparative column (100 Å, 5 μm, 10 × 250 mm) and a flow rate of 2 mL min^−1^. The mobile phase consisted of two solutions: H_2_O with 0.1% TFA and MeCN with 0.1% TFA, run in a gradient from 95% H_2_O/5% MeCN to 100% MeCN over 60 min. Analytical HPLC utilised a Waters Sunfire C18 analytical column (100 Å, 5 μm, 4.6 × 250 mm) and a flow rate of 0.2 mL min^−1^. The mobile phase consisted of two solvent mixtures: H_2_O with 0.1% TFA and MeCN with 0.1% TFA, run in a gradient from 100% H_2_O to 100% MeCN over 45 min.

The log*P* values of the Gd(III) complexes were measured by means of a standard reverse-phase HPLC method on a Waters 2695 separations module equipped with the Waters Alliance column heater (set at 30 °C) and Waters 2996 Photodiode Array (PDA) detector using a Waters Sunfire C18 column (100 Å, 5 μm, 2.1 × 150 mm). The mobile phase consisted of 65% (*v/v*) MeOH in a 50 mM sodium phosphate buffer adjusted to pH 7.4 by mixing 50 mM solutions of Na_2_HPO_4_ and NaH_2_PO_4_. The samples, along with a set of standards with known log*P* values, were determined using the solvent with an isocratic flow-rate of 0.2 mL min^−1^. The retention times of acetone (log*D*_7.4_ − 0.24), aniline (log*D*_7.4_ 0.90), phenol (log*D*_7.4_ 1.50), toluene (log*D*_7.4_ 2.730), cumene (log*D*_7.4_ 3.66), triphenylamine (log*D*_7.4_ 5.74), and hexachlorobenzene (log*D*_7.4_ 6.35) were plotted against their literature log*D*_7.4_ values, adjusting for the T_0_, to establish a calibration curve^42^. Fitting an exponential curve to the dataset generated the equation (y = 0.8822ln(x) + 1.8164; *r*^2^ = 0.984) from which the sample log*P* values could be derived using their retention times. The HPLC experiments were performed in triplicate.

### Synthetic methods

1,4,7,10-Tetraazacyclododecane-1,4,7-tris(*tert*-butyl acetate) (DO3A-^*t*^Bu_3_·HBr) was prepared using a method adapted from Moore et al.^9,43–45^. 1,4,7,10-Tetraazacyclododecane-1,4,7-tris(*tert*-butyl acetate)-10-acetic acid (DOTA-^*t*^Bu_3_) was prepared using a method adapted from Strauch^46^. Complex **11** was prepared as previously described by Morrison et al^8^.

#### General phosphonium salt procedure (GP1)

Phosphonium salts of interest were prepared by means of dropwise addition of the precursor arylphosphines in toluene to a stirred solution of the relevant dibromo-*p*-xylyl, alkyl or TEG linkers in toluene, followed by heating at reflux (unless specified). The crystallized product was filtered off and washed with toluene and diethyl ether to yield the phosphonium salt in high purity.

#### General DOTA linking procedure (GP2)

The bromine functional groups of the phosphonium salts prepared in GP1 were replaced with azide groups by stirring with NaN_3_ in DMF at RT for 72 h. The DMF was removed under a flow of N_2_, and the residue was then dissolved in CHCl_3_ and any excess NaN_3_ filtered off. The filtrate was reduced *in vacuo* to yield the phosphonium azide in high purity (> 95%). The azide group was reduced to an amine using 10 mol% Pd/C stirred in MeOH under a H_2_ atmosphere at RT for 6 h. The catalyst was removed by means of filtration, and the solvent volume reduced to afford the phosphonium-amine in high purity. The phosphonium targeting vectors were coupled to the DOTA chelating component by means of a peptide coupling reaction performed in peptide-grade DMF with the HATU coupling reagent in the presence of excess NMM and stirred at RT for 24 h. The DMF and NMM were removed under N_2_, and the residue was deprotected by the addition of neat TFA upon stirring the mixture for a further 12 h at RT. The volatiles were removed *in vacuo*, and the crude residue dissolved in H_2_O and washed with CHCl_3_. The aqueous fraction was reduced *in vacuo* to give a hygroscopic solid which was purified by means of reverse-phase HPLC, and the product lyophilised to afford the pure ligand.

#### General complexation procedure (GP3)

The purified ligands were complexed with Gd(III) by stirring a suspension of excess Gd_2_O_3_ in a minimum amount of H_2_O for 72 h at RT. Any remaining Gd_2_O_3_ was removed by means of centrifugation and subsequent filtration through a 0.2 μm filter. The filtrate was lyophilised to afford the purified Gd(III) complex as a colourless powder.

2,2′,2″-(10-(2-((4-((Triphenylphosphonio)methyl)benzyl)amino)-2-oxoethyl)-1,4,7,10-tetraazacyclododecane-1,4,7-triyl)triacetatogadolinium(III) trifluoroacetate (**1**): *GP1* using triphenylphosphine (2.64 g, 10.07 mmol) and α*,*α*’*-dibromo-*p*-xylene (2.74 g, 10.38 mmol) in 30 mL of toluene heated at reflux for 4 h to yield 5.02 g (95.3%) of the phosphonium salt. By following *GP2*, the phosphonium salt (5.02 g, 9.55 mmol) was added to excess NaN_3_ (1.87 g, 28.74 mmol) in DMF (50 mL) affording the colourless, crystalline azide (4.4 g, 94.4%). Hydrogenation of the azide (513 mg, 1.05 mmol) using 10% Pd/C (*ca.* 50 mg) in MeOH (20 mL) under a H_2_ atmosphere yielded the corresponding amine (444 mg, 91.4%) as a colourless solid. Slow addition of this amine (742 mg, 1.61 mmol) in DMF (20 mL) to a stirred solution of DOTA-^*t*^Bu_3_ (942 mg, 1.65 mmol), HATU (751 mg, 1.97 mmol), and NMM (720 μL, 662 mg, 6.55 mmol) in DMF (40 mL) yielded the crude protected ligand, which was subsequently deprotected with neat TFA (10 mL), dissolved in H_2_O (100 mL), washed, purified by means of reverse-phase HPLC, and lyophilised to afford the pure ligand as a colourless powder (1.0 g, 73.1%). ESI–MS for [M^+^]: calculated *m/z* 768.35, observed *m/z* 768.32. ^1^H NMR (D_2_O) δ 7.85–7.80 (m, 3H, Ar, *p*-PhP^+^), 7.61–7.54 (m, 12H, Ar, *o*-PhP^+^, *m*-PhP^+^), 7.12 (d, 2H, Ar, *o*-xy, ^3^*J*_HH_ = 7.9 Hz), 6.92 (d, 2H, Ar, *m*-xy, ^3^*J*_HH_ = 8.2 Hz), 4.33 (br s, 2H, CH_2_P^+^), 4.20–3.00 [m, 26H: 3.99 (br s, 2H, xyCH_2_N), 3.66 (br s, 2H, NCH_2_C(O)), 3.37 (br s, 6H, NCH_2_C(O)O), 3.21 (br s, 16H, NCH_2_CH_2_N)]. ^13^C NMR (D_2_O) δ 162.7 (q, C(O) TFA, ^2^*J*_CF_ = 35.7 Hz), 138.2 (s, Ar, *p*-xy), 135.1 (d, Ar, *p*-PhP^+^, ^4^*J*_CP_ = 2.0 Hz), 134.0 (d, Ar, *m*-PhP^+^, ^3^*J*_CP_ = 9.8 Hz), 131.2 (d, Ar, *o*-xy, ^3^*J*_CP_ = 4.5 Hz), 129.9 (d, Ar, *o*-PhP^+^, ^2^*J*_CP_ = 12.8 Hz), 127.7 (d, Ar, *m*-xy, ^4^*J*_CP_ = 1.6 Hz), 126.5 (d, Ar, *i*-xy, ^2^*J*_CP_ = 8.3 Hz), 117.2 (m), 54.9, 53.3, 50.8–48.9 (br m, NCH_2_CH_2_N), 42.6 (s,CH_2_NH), 29.4 (d, ^+^PCH_2_, ^1^*J*_CP_ = 48.3 Hz), C(O) of the acetate arms could not be located. ^31^P{^1^H} NMR (D_2_O) δ 22.1 (s). The purified ligand (0.96 g, 1.09 mmol) was stirred with a suspension of Gd_2_O_3_ (0.30 g, 0.83 mmol) in water for 72 h at RT, and excess Gd_2_O_3_ removed and the solution lyophilised to afford the pure complex as a colourless powder (1.1 g, 97.4%). Purity > 95% by HPLC. ESI-FT-ICR-MS for [M]^+^: calculated *m/z* 923.25269, observed *m/z* 923.25249.

2,2′,2″-(10-(2-((4-((Methyldiphenylphosphonio)methyl)benzyl)amino)-2-oxoethyl)-1,4,7,10-tetraazacyclododecane-1,4,7-triyl)triacetatogadolinium(III) trifluoroacetate (**2**): *GP1* using methyldiphenylphosphine (1.00 mL, 1.08 g, 5.37 mmol) and α*,*α*’*-dibromo-*p*-xylene (1.60 g, 6.06 mmol) each in 20 mL of toluene at reflux for 2 h to yield 2.0 g (81.4%) of the phosphonium salt. Following *GP2*, the phosphonium salt (384 mg, 0.83 mmol) was added to excess NaN_3_ (177 mg, 2.72 mmol) in DMF (15 mL) affording the colourless crystalline azide (344 mg, 97.6%). Hydrogenation of the azide (1.01 g, 2.37 mmol) using 10% Pd/C (*ca.* 100 mg) in MeOH (50 mL) under a H_2_ atmosphere yielded the corresponding amine (793 mg, 83.6%) as a colourless solid. Slow addition of this amine (770 mg, 1.92 mmol) in DMF (10 mL) to a stirred solution of DOTA-^*t*^Bu_3_ (1.08 g, 1.89 mmol), HATU (864 mg, 2.27 mmol), and NMM (830 μL, 764 mg, 7.55 mmol) in DMF (30 mL) yielded the crude protected ligand, which was subsequently deprotected with neat TFA (12 mL), dissolved in H_2_O (100 mL), washed, purified by means of reverse-phase HPLC, and lyophilised to afford the pure ligand as a colourless powder (1.1 g, 72.5%). ESI–MS for [M^+^·^*t*^Bu_3_]: calculated *m/z* 874.52, observed *m/z* 874.55. ESI–MS for [M^+^]: calculated *m/z* 706.34, observed *m/z* 706.34. ^1^H NMR (CDCl_3_) δ 7.81–7.70 (m, 4H, Ar, *o*-PhP^+^), 7.62–7.60 (m, 2H, Ar, *p*-PhP^+^), 7.54–7.49 (m, 4H, Ar, *m*-PhP^+^), 7.21–7.16 (m, 2H, Ar, *o*-xy, ^3^*J*_HH_ = 8.1 Hz), 7.09 (d, 2H, Ar, *m*-xy, ^3^*J*_HH_ = 8.0 Hz), 4.44 (br s, 2H, ^+^PCH_2_), 4.25–3.11 [m, 26H: 4.09 (br s, 2H, xyCH_2_N), 3.70 (br s, 2H, NCH_2_C(O)), 3.42 (br s, 6H, NCH_2_C(O)O), 3.24 (br s, 16H, NCH_2_CH_2_N)], 2.71 (d, 3H, ^+^PCH_3_, ^2^*J*_HP_ = 15.2 Hz). ^13^C NMR (D_2_O) δ 166.1 (q, C(O) TFA, ^2^*J*_CF_ = 36.3 Hz), 138.4 (s, Ar, *p*-xy), 135.2 (d, Ar, *p*-PhP^+^, ^4^*J*_CP_ = 1.8 Hz), 133.6 (d, Ar, *m*-PhP^+^, ^3^*J*_CP_ = 9.1 Hz), 130.9 (d, Ar, *o*-xy, ^3^*J*_CP_ = 5.1 Hz), 129.9 (d, Ar, *o*-PhP^+^, ^2^*J*_CP_ = 12.7 Hz), 127.4 (s, Ar, *m*-xy), 126.7 (d, Ar, *i*-xy, ^2^*J*_CP_ = 9.2 Hz), 117.9 (m), 54.7, 53.0, 51.4–48.3 (br m, NCH_2_CH_2_N), 42.1 (s, CH_2_NH_2_), 29.9 (d, ^+^PCH_2_, ^1^*J*_CP_ = 48.3 Hz), 5.4 (d, ^+^PCH_3_, ^1^*J*_CP_ = 57.0 Hz), C(O) of the acetate arms could not be located. ^31^P{^1^H} NMR ((D_2_O) δ 22.2 (s). *GP3* The purified ligand (1.04 g, 1.27 mmol) was stirred with Gd_2_O_3_ (352 mg, 0.97 mmol) in water, excess Gd_2_O_3_ removed and the solution lyophilised to afford the pure complex as a colourless powder (1.2 g, 94.3%). Purity > 95% by HPLC. ESI-FT-ICR-MS for [M]^+^: calculated *m/z* 861.23704, observed *m/z* 861.23663.

2,2′,2″-(10-(2-((4-((Isopropyldiphenylphosphonio)methyl)benzyl)amino)-2-oxoethyl)-1,4,7,10-tetraazacyclododecane-1,4,7-triyl)triacetatogadolinium(III) trifluoroacetate (**3**): *GP1* using isopropyldiphenylphosphine (1.04 g, 4.56 mmol) and α*,*α*’*-dibromo-*p*-xylene (1.40 g, 5.31 mmol) each in 20 mL of toluene at RT for 24 h, ensuring the air sensitive phosphine precursor was kept out of contact with oxygen, to yield 1.7 g (73.9%) of the phosphonium salt. The phosphonium salt (559 mg, 0.91 mmol) was added to excess NaN_3_ (207 mg, 3.18 mmol) in DMF (15 mL) affording the colourless crystalline azide (508 mg, 96.8%). Hydrogenation of the azide (596 mg, 1.31 mmol) using 10% Pd/C (*ca.* 60 mg) in MeOH (20 mL) under a H_2_ atmosphere yielded the corresponding amine (506 mg (89.9%) as a colourless solid. Slow addition of this amine (468 mg, 1.09 mmol) in DMF (5 mL) to a stirred solution of DOTA-^*t*^Bu_3_ (647 mg, 1.13 mmol), HATU (522 mg, 1.37 mmol), and NMM (504 μL, 464 mg, 4.58 mmol) in DMF (15 mL) yielded the crude protected ligand, which was subsequently deprotected with neat TFA (10 mL), dissolved in H_2_O (100 mL), washed, purified by means of reverse-phase HPLC, and lyophilised to afford the pure ligand as a colourless powder (541 mg, 58.4%). ESI–MS for [M^+^·^*t*^Bu_3_]: calculated *m/z* 902.56, observed *m/z* 902.51. ESI–MS for [M^+^]: calculated *m/z* 734.37, observed *m/z* 734.40. ^1^H NMR (D_2_O) δ 7.71 (m, 2H, Ar, *p*-PhP^+^), 7.60–7.45 (m, 8H, Ar, *o*-PhP^+^, *m*-PhP^+^), 6.99 (d, 2H, Ar, *m*-xy, ^3^*J*_HH_ = 7.2 Hz), 6.79 (br d, 2H, Ar, *o*-xy, ^3^*J*_HH_ = 7.9 Hz), 4.25 (d, 2H, ^+^PCH_2_, ^2^*J*_HP_ = 14.6 Hz), 4.10–2.7 [m, 26H: 4.02 (br s, 2H, CH_2_NH_2_), 3.57 (br s, 2H, NCH_2_C(O)), 3.44 (br s, 1H, ^+^PCH), 3.32 (br s, 6H, NCH_2_C(O)O), 2.91 (br s, 16H, NCH_2_CH_2_N)] 1.19 (dd, 6H, ^+^PCH(CH_3_)_2_, ^3^*J*_HP_ = 18.2 Hz, ^3^*J*_HH_ = 7.1 Hz). ^13^C NMR (D_2_O) δ 168.1 (q, C(O) TFA, ^2^*J*_CF_ = 36.6 Hz), 138.6 (d, Ar, *p*-xy, ^5^*J*_CP_ = 1.9 Hz), 135.1 (d, Ar, *p*-PhP^+^, ^4^*J*_CP_ = 2.9 Hz), 134.1 (d, Ar, *o*-PhP^+^, ^2^*J*_CP_ = 8.6 Hz), 131.5 (d, Ar, *o*-xy, ^3^*J*_CP_ = 4.7 Hz), 129.7 (d, Ar, *m*-PhP^+^, ^3^*J*_CP_ = 12.5 Hz), 126.5 (d, Ar, *m*-xy, ^4^*J*_CP_ = 2.5 Hz), 123.7 (m), 116.1 (m), 56.6, 53.3, 51.8–47.0 (br m, NCH_2_CH_2_N), 42.3 (s, CH_2_NH_2_), 27.3 (d, ^+^PCH_2_, ^1^*J*_CP_ = 46.8 Hz), 20.3 (d, ^+^PCH, ^1^*J*_CP_ = 47.3 Hz), 15.2 (d), C(O) of the acetate arms could not be located. ^31^P{^1^H} NMR (D_2_O) δ 34.0 (s). *GP3* The purified ligand (505 mg, 596 μmol) was stirred with Gd_2_O_3_ (170 mg, 469 μmol) in water, excess Gd_2_O_3_ removed and the solution lyophilised to afford the pure complex as a colourless powder (576 mg, 93.2%). Purity > 95% by HPLC. ESI-FT-ICR-MS for [M]^+^: calculated *m/z* 889.26927, observed *m/z* 889.26805.

2,2′,2″-(10-(2-((4-((Diphenyl(p-tolyl)phosphonio)methyl)benzyl)amino)-2-oxoethyl)-1,4,7,10-tetraazacyclododecane-1,4,7-triyl)triacetatogadolinium(III) trifluoroacetate (**4**): *GP1* using diphenyl(*p-*tolyl)phosphine (599 mg, 2.17 mmol) and α*,*α*’*-dibromo-*p*-xylene (653 mg, 2.47 mmol) each in 20 mL of toluene at RT for 24 h to yield 1.0 g (89.4%) of the phosphonium salt. The phosphonium salt (1.14 g, 2.12 mmol) was added to excess NaN_3_ (496 mg, 7.63 mmol) in DMF (20 mL) affording the colourless, crystalline azide (983 mg, 92.4%). Hydrogenation of the azide (330 mg, 0.66 mmol) using 10% Pd/C (*ca.* 30 mg) in MeOH (15 mL) under a H_2_ atmosphere yielded the corresponding amine (276 mg (88.1%) as a colourless solid. Slow addition of this amine (137 mg, 228 μmol) in DMF (5 mL) to a stirred solution of DOTA-^*t*^Bu_3_ (135 mg, 235 μmol), HATU (112 mg, 296 μmol), and NMM (110 μL, 101 mg, 1.00 mmol) in DMF (10 mL) yielded the crude protected ligand, which was subsequently deprotected with neat TFA (5 mL), dissolved in H_2_O (100 mL), washed, purified by means of reversed-phase HPLC, and lyophilised to afford the pure ligand as a colourless powder (183 mg, 71.0%). ESI–MS for [M·^*t*^Bu_3_ + Na]^2+^: calculated *m/z* 486.77, observed *m/z* 486.75. ESI–MS for [M^+^]: calculated *m/z* 782.37, observed *m/z* 782.32. ^1^H NMR (D_2_O) δ 7.90–7.88 (m, 2H, Ar, *p*-PhP^+^), 7.68–7.61 (m, 8H, Ar, *o*-PhP^+^, *m*-PhP^+^), 7.61–7.52 (m, 4H, Ar, *o*-Ph(Me)P^+^, *m*-Ph(Me)P^+^), 7.48 (d, 2H, Ar, *m*-xy, ^3^*J*_HH_ = 8.4 Hz), 7.05 (d, 2H, Ar, *o*-xy, ^3^*J*_HH_ = 8.3 Hz,), 4.15 (d, 2H, CH_2_P^+^, ^2^*J*_HP_ = 15.3 Hz), 3.95–3.02 [m, 26H: 3.87 (br s, 2H, xyCH_2_N), 3.60 (br s, 2H, NCH_2_C(O)), 3.35 (br s, 6H, NCH_2_C(O)O), 3.21 (br s, 16H, NCH_2_CH_2_N)], 2.49 (s, 3H, CH_3_). ^13^C NMR (D_2_O) δ 162.8 (q, C(O) TFA, ^2^*J*_CF_ = 35.7 Hz), 147.1 (s, Ar, *p*-Ph(Me)P^+^), 135.2 (s, Ar, *p*-xy, *p*-PhP^+^), 133.7 (d, Ar, *m*-PhP^+^, *m*-Ph(Me)P^+^, ^3^*J*_CP_ = 9.1 Hz), 132.0 (br s, Ar, *o*-xy), 131.4 (d, Ar, *o*-Ph(Me)P^+^, ^2^*J*_CP_ = 12.8 Hz), 130.7 (m, Ar, *o*-PhP^+^), 129.3–129.1 (m, Ar, *m*-xy, *i*-xy), 116.6 (m), 112.8 (m), 55.2, 53.5, 51.2–49.7 (br m, NCH_2_CH_2_N), 48.4 (s, CH_2_NH), 29.4 (d, ^+^PCH_2_, ^1^*J*_CP_ = 48.3 Hz), 20.9 (s, CH_3_), C(O) of the acetate arms could not be located. ^31^P{^1^H} NMR (D_2_O) δ 22.0 (s). *GP3* The purified ligand (172 mg, 192 μmol) was stirred with Gd_2_O_3_ (55 mg, 152 μmol) in water, excess Gd_2_O_3_ removed and the solution lyophilised to afford the pure complex as a colourless powder (191 mg, 91.7%). Purity > 95% by HPLC. ESI-FT-ICR-MS for [M]^+^: calculated *m/z* 937.26834, observed *m/z* 937.26658.

2,2′,2″-(10-(2-Oxo-2-((4-((tris(4-methoxyphenyl)phosphonio)methyl)benzyl)amino)ethyl)-1,4,7,10-tetraazacyclododecane-1,4,7-triyl)triacetatogadolinium(III) trifluoroacetate (**5**): *GP1* using tris(4-methoxyphenyl)phosphine (1.01 g, 2.87 mmol) and α*,*α*’*-dibromo-*p*-xylene (892 mg, 3.38 mmol) each in 20 mL of toluene at RT for 24 h to yield 1.4 g (81.4%) of the phosphonium salt. The phosphonium salt (1.02 g, 1.66 mmol) was added to excess NaN_3_ (393 mg, 6.04 mmol) in DMF (22 mL) affording the colourless crystalline azide (928 mg, 96.8%). Hydrogenation of the azide (582 mg, 1.01 mmol) using 10% Pd/C (*ca.* 60 mg) in MeOH (25 mL) under a H_2_ atmosphere yielded the corresponding amine (481 mg, 86.5%) as a colourless solid. Slow addition of this amine (530 mg, 0.96 mmol) in DMF (10 mL) to a stirred solution of DOTA-^*t*^Bu_3_ (597 mg, 1.04 mmol), HATU (486 mg, 1.28 mmol), and NMM (470 μL, 432 mg, 4.27 mmol) in DMF (20 mL) yielded the crude protected ligand, which was subsequently deprotected with neat TFA (10 mL), dissolved in H_2_O (100 mL), washed, purified by means of reverse-phase HPLC, and lyophilised to afford the pure ligand as a colourless powder (637 mg, 68.3%). ESI–MS for [M^+^·^*t*^Bu_3_]: calculated *m/z* 1026.57, observed *m/z* 1026.52. ESI–MS for [M^+^]: calculated *m/z* 858.38, observed *m/z* 858.35. ^1^H NMR (D_2_O) δ 7.52 (dd, 6H, Ar, *o*-PhP^+^, ^3^*J*_HH_ = 9.1 Hz, ^3^*J*_HP_ = 11.9 Hz), 7.25 (dd, 6H, Ar, *m*-PhP^+^, ^3^*J*_HH_ = 8.6 Hz, ^4^*J*_HP_ = 1.7 Hz), 7.12 (d, 2H, Ar, *m*-xy, ^3^*J*_HH_ = 8.0 Hz, ^5^*J*_HP_ = 2.0 Hz), 7.00 (d, 2H, Ar, *o*-xy, ^3^*J*_HH_ = 8.3 Hz, ^4^*J*_HP_ = 2.1 Hz), 4.50 (d, 2H, ^+^PCH_2_, ^2^*J*_HP_ = 14.8 Hz), 4.23 (s, 2H, xyCH_2_N), 3.95–2.73 [m, 33H: 3.86 (s, 9H, CH_3_), 3.61 (br s, 2H, NCH_2_C(O)), 3.37 (br s, 6H, NCH_2_C(O)O), 3.13 (br s, 16H, NCH_2_CH_2_N)]. ^13^C NMR (D_2_O) δ 164.4 (d, Ar, *p*-PhP^+^, ^4^*J*_CP_ = 2.4 Hz), 162.9 (q, C(O) TFA, ^2^*J*_CF_ = 34.8 Hz), 136.7 (d, *p*-xy, ^5^*J*_CP_ = 3.1 Hz), 135.7 (d, Ar, *m*-PhP^+^, ^3^*J*_CP_ = 11.3 Hz), 132.0 (d, Ar, *o*-xy, ^3^*J*_CP_ = 4.7 Hz), 131.3 (d, Ar, *m*-xy, ^4^*J*_CP_ = 2.2 Hz), 116.3 (m), 115.6 (d, Ar, *o*-PhP^+^, ^2^*J*_CP_ = 13.5 Hz), 108.1 (d), 55.8 (s, CH_3_), 54.7, 53.4, 50.8–48.9 (br m, NCH_2_CH_2_N), 43.6 (s, CH_2_N), 30.4 (d, ^+^PCH_2_, ^1^*J*_CP_ = 49.7 Hz), C(O) of the acetate arms could not be located. ^31^P{^1^H} NMR (D_2_O) δ 20.4 (s). *GP3* The purified ligand (592 mg, 609 μmol) was stirred with Gd_2_O_3_ (175 mg, 483 μmol) in water, excess Gd_2_O_3_ removed and the solution lyophilised to afford the pure complex as a colourless powder (648 mg, 91.6%). Purity > 95% by HPLC. ESI-FT-ICR-MS for [M]^+^: calculated *m/z* 1013.28438, observed *m/z* 1013.28328.

2,2′,2″-(10-(2-Oxo-2-((3-(triphenylphosphonio)propyl)amino)ethyl)-1,4,7,10-tetraazacyclododecane-1,4,7-triyl)triacetato gadolinium(III) trifluoroacetate (**6**): The phosphonium salt (1.41 g, 3.03 mmol) was added to excess NaN_3_ (592 mg, 9.11 mmol) in DMF (25 mL) affording the colourless crystalline azide (1.14 g, 88.5%). Hydrogenation of the azide (300 mg, 0.70 mmol) using 10% Pd/C (*ca.* 30 mg) in MeOH (20 mL) under a H_2_ atmosphere yielded the corresponding amine (235 mg, 83.5%) as a colourless solid. Slow addition of this amine (275 mg, 0.69 mmol) in DMF (10 mL) to a stirred solution of DOTA-^*t*^Bu_3_ (408 mg, 0.71 mmol), HATU (332 mg, 0.87 mmol), and NMM (325 μL, 299 mg, 2.96 mmol) in DMF (15 mL) yielded the crude protected ligand, which was subsequently deprotected with neat TFA (10 mL), dissolved in H_2_O (100 mL), washed, purified by means of reverse-phase HPLC, and lyophilised to afford the pure ligand as a colourless powder (461 mg, 81.8%). MALDI-TOF–MS for [M^+^·^*t*^Bu_3_]: calculated *m/z* 874.52, observed *m/z* 874.55. MALDI-TOF–MS for [M^+^]: calculated *m/z* 706.34, observed *m/z* 706.40.^1^H NMR (D_2_O) δ 7.81–7.53 [m, 15H: 7.76–7.70 (m, 3H, Ar, *p*-PhP^+^), 7.66–7.55 (m, 12H, Ar, *o*-PhP^+^, *m*-PhP^+^, ^3^*J*_HH_ = 7.9 Hz,)], 3.85 (m, 2H, ^+^PCH_2_, ^2^*J*_HP_ = 16.3 Hz, ^3^*J*_HH_ = 8.4 Hz), 3.57–3.12 [m, 26H: 3.61 (br s, 2H, NCH_2_C(O)), 3.32 (br s, 6H, NCH_2_C(O)O), 3.18 (br s, 18H, NCH_2_CH_2_N, **X**-CH_2_N)], 1.56 (m, 2H, 2-CH_2_). ^13^C NMR (D_2_O) δ 162.7, 134.3 (m, Ar, *p*-PhP^+^), 133.1 (d, Ar, *m*-PhP^+^, ^3^*J*_CP_ = 9.6 Hz), 129.8 (d, Ar, *o*-PhP^+^, ^2^*J*_CP_ = 12.5 Hz), 118.1 (m), 57.3, 56.4, 50.4–48.7 (br m, NCH_2_CH_2_N), 42.8 (d, 3-CH_2_N, ^3^*J*_CP_ = 16.3 Hz), 24.9 (s, 2-CH_2_), 19.9 (d, ^+^PCH_2_, ^1^*J*_CP_ = 55.1 Hz). ^31^P{^1^H} NMR (D_2_O) δ 24.0 (s). The purified ligand (430 mg, 525 μmol) was stirred with Gd_2_O_3_ (153 mg, 422 μmol) in water, excess Gd_2_O_3_ removed and the solution lyophilised to afford the pure complex as a colourless powder (469 mg, 88.6%). Purity > 95% by HPLC. MALDI-TOF–MS for [M]^+^: calculated *m/z* 861.237, observed *m/z* 861.317.

2,2′,2″-(10-(2-Oxo-2-((4-(triphenylphosphonio)butyl)amino)ethyl)-1,4,7,10-tetraazacyclododecane-1,4,7-triyl)triacetato gadolinium(III) trifluoroacetate (**7**): The phosphonium salt (1.68 g, 3.50 mmol) was added to excess NaN_3_ (695 mg, 10.69 mmol) in DMF (25 mL) affording the colourless crystalline azide (1.4 g, 93.3%). Hydrogenation of the azide (460 mg, 1.05 mmol) using 10% Pd/C (*ca.* 50 mg) in MeOH (20 mL) under a H_2_ atmosphere yielded the corresponding amine (383 mg, 88.4%) as a colourless solid. Slow addition of this amine (365 mg, 0.88 mmol) in DMF (10 mL) to a stirred solution of DOTA-^*t*^Bu_3_ (520 mg, 0.91 mmol), HATU (427 mg, 1.12 mmol), and NMM (400 μL, 368 mg, 3.64 mmol) in DMF (20 mL) yielded the crude protected ligand, which was subsequently deprotected with neat TFA (15 mL), dissolved in H_2_O (100 mL), washed, purified by means of reverse-phase HPLC, and lyophilised to afford the pure ligand as a colourless powder (312 mg, 42.5%). MALDI-TOF–MS for [M^+^·^*t*^Bu_3_]: calculated *m/z* 888.54, observed *m/z* 888.57. MALDI-TOF–MS for [M^+^]: calculated *m/z* 720.35, observed *m/z* 720.32. ^1^H NMR (D_2_O) δ 7.85–7.49 [m, 15H: 7.81–7.73 (m, 3H, Ar, *p*-PhP^+^), 7.68–7.57 (m, 12H, Ar, *o*-PhP^+^, *m*-PhP^+^, ^3^*J*_HH_ = 8.1 Hz,)], 3.83 (m, 2H, ^+^PCH_2_, ^2^*J*_HP_ = 14.7 Hz), 3.62–3.09 [m, 26H: 3.67 (br s, 2H, NCH_2_C(O)), 3.28 (br s, 6H, NCH_2_C(O)O), 3.13 (br s, 18H, NCH_2_CH_2_N, 4-CH_2_N)], 1.77 (q_i_, 2H, 3-CH_2_^3^*J*_HH_ = 8.2 Hz), 1.57 (dq_i_, 2H, 2-CH_2_, ^3^*J*_HH_ = 8.2 Hz, ^3^*J*_HP_ = 6.1 Hz). ^13^C NMR (D_2_O) δ 162.6, 134.9 (d, Ar, *p*-PhP^+^), 133.4 (d, Ar, *m*-PhP^+^, ^3^*J*_CP_ = 9.9 Hz), 130.3 (d, Ar, *o*-PhP^+^, ^2^*J*_CP_ = 12.3 Hz), 117.8 (m), 57.3, 56.4, 50.4–48.7 (br m, NCH_2_CH_2_N), 41.4 (s, 4-CH_2_N, ^3^*J*_CP_ = 14.0 Hz), 31.3 (d, 3-CH_2_, ^3^*J*_CP_ = 12.3 Hz), 22.4 (d, ^+^PCH_2_, ^1^*J*_CP_ = 51.8 Hz), 19.4 (s, 2-CH_2_). ^31^P{^1^H} NMR (D_2_O) δ 23.9 (s). The purified ligand (284 mg, 341 μmol) was stirred with Gd_2_O_3_ (98 mg, 270 μmol) in water, excess Gd_2_O_3_ removed and the solution lyophilised to afford the pure complex as a colourless powder (342 mg, 97.9%). Purity > 95% by HPLC. MALDI-TOF–MS for [M]^+^: calculated *m/z* 875.253, observed *m/z* 875.213.

2,2′,2″-(10-(2-Oxo-2-((5-(triphenylphosphonio)pentyl)amino)ethyl)-1,4,7,10-tetraazacyclododecane-1,4,7-triyl)triacetato gadolinium(III) trifluoroacetate (**8**): The phosphonium salt (1.96 g, 3.98 mmol) was added to excess NaN_3_ (805 mg, 12.38 mmol) in DMF (16 mL) affording the colourless crystalline azide (1.6 g, 91.2%). Hydrogenation of the azide (614 mg, 1.05 mmol) using 10% Pd/C (*ca.* 60 mg) in MeOH (20 mL) under a H_2_ atmosphere yielded the corresponding amine (504 mg, 87.2%) as a colourless solid. Slow addition of this amine (372 mg, 0.87 mmol) in DMF (10 mL) to a stirred solution of DOTA-^*t*^Bu_3_ (506 mg, 0.88 mmol), HATU (408 mg, 1.07 mmol), and NMM (380 μL, 350 mg, 3.46 mmol) in DMF (20 mL) yielded the crude protected ligand, which was subsequently deprotected with neat TFA (15 mL), dissolved in H_2_O (100 mL), washed, purified by means of reverse-phase HPLC, and lyophilised to afford the pure ligand as a colourless powder (421 mg, 57.2%). MALDI-TOF–MS for [M^+^·^*t*^Bu_3_]: calculated *m/z* 902.56, observed *m/z* 902.52. MALDI-TOF–MS for [M^+^]: calculated *m/z* 734.37, observed *m/z* 734.37. ^1^H NMR (D_2_O) δ 7.72–7.56 (m, 15H, Ar, *o*-PhP^+^, *p*-PhP^+^, *m*-PhP^+^), 3.65 (m, 2H, ^+^PCH_2_, ^2^*J*_HP_ = 12.3 Hz), 3.55–3.03 [m, 26H: 3.53 (br s, 2H, NCH_2_C(O)), 3.30 (br s, 6H, NCH_2_C(O)O), 3.11 (br s, 18H, NCH_2_CH_2_N, 5-CH_2_N)], 1.65 (q_i_, 2H, 4-CH_2,_^3^*J*_HH_ = 7.8 Hz), 1.55 (m, 2H, 2-CH_2_), 1.51 (m, 2H, 3-CH_2_). ^13^C NMR (D_2_O) δ 162.7, 135.7 (d, Ar, *p*-PhP^+^), 133.9 (d, Ar, *m*-PhP^+^, ^3^*J*_CP_ = 8.9 Hz), 130.8 (d, Ar, *o*-PhP^+^, ^2^*J*_CP_ = 12.0 Hz), 117.4 (m), 58.8, 57.2, 51.6–50.2 (br m, NCH_2_CH_2_N), 43.8 (s, 5-CH_2_N), 32.2 (s 4-CH_2_), 24.3 (d, ^+^PCH_2_, ^1^*J*_CP_ = 51.9 Hz), 20.1 (s, 3-CH_2_), 19.5 (s, 2-CH_2_). ^31^P{^1^H} NMR (D_2_O) δ 24.3 (s). *GP3* The purified ligand (386 mg, 455 μmol) was stirred with Gd_2_O_3_ (124 mg, 341 μmol) in water, excess Gd_2_O_3_ removed and the solution lyophilised to afford the pure complex as a colourless powder (452 mg, 95.7%). Purity > 95% by HPLC. MALDI-TOF–MS for [M]^+^: calculated *m/z* 889.268, observed *m/z* 889.267.

2,2′,2″-(10-(2-Oxo-2-((6-(triphenylphosphonio)hexyl)amino)ethyl)-1,4,7,10-tetraazacyclododecane-1,4,7-triyl)triacetato gadolinium(III) trifluoroacetate (**9**): The phosphonium salt (2.44 g, 4.83 mmol) was added to excess NaN_3_ (962 mg, 14.80 mmol) in DMF (25 mL) affording the colourless crystalline azide (1.9 g, 85.6%). Hydrogenation of the azide (430 mg, 0.92 mmol) using 10% Pd/C (*ca.* 40 mg) in MeOH (20 mL) under a H_2_ atmosphere yielded the corresponding amine (377 mg, 92.8%) as a colourless solid. Slow addition of this amine (362 mg, 0.82 mmol) in DMF (10 mL) to a stirred solution of DOTA-^*t*^Bu_3_ (472 mg, 0.82 mmol), HATU (401 mg, 1.05 mmol), and NMM (360 μL, 331 mg, 3.27 mmol) in DMF (20 mL) yielded the crude protected ligand, which was subsequently deprotected with neat TFA (10 mL), dissolved in H_2_O (100 mL), washed, purified by means of reverse-phase HPLC, and lyophilised to afford the pure ligand as a colourless powder (347 mg, 49.2%). MALDI-TOF–MS for [M^+^·^*t*^Bu_3_]: calculated *m/z* 916.57, observed *m/z* 916.55. MALDI-TOF–MS for [M^+^]: calculated *m/z* 748.38, observed *m/z* 748.41. ^1^H NMR (D_2_O) δ 7.82–7.51 [m, 15H: 7.77–7.65 (m, 9H, Ar, *o*-PhP^+^, *p*-PhP^+^), 7.61–7.54 (m, 6H, Ar, *m*-PhP^+^)], 3.44 (m, 2H, ^+^PCH_2_, ^2^*J*_HP_ = 12.3 Hz), 3.38–3.02 [m, 26H: 3.31 (br s, 2H, NCH_2_C(O)), 3.20 (br s, 6H, NCH_2_C(O)O), 3.09 (br s, 18H, NCH_2_CH_2_N, 6-CH_2_N)], 1.62 (br m, 2H, 5-CH_2_), 1.41 (br m, 2H, 2-CH_2_), 1.24 (br m, 2H, 4-CH_2_), 1.17 (br m, 2H, 3-CH_2_). ^13^C NMR (D_2_O) δ 162.7, 134.4 (d, Ar, *p*-PhP^+^), 132.9 (d, Ar, *m*-PhP^+^, ^3^*J*_CP_ = 9.3 Hz), 129.7 (d, Ar, *o*-PhP^+^, ^2^*J*_CP_ = 11.8 Hz), 117.0 (m), 58.1, 56.8, 51.4–48.3 (m, NCH_2_CH_2_N), 42.8 (s, 6-CH_2_N), 32.4 (s, 5-CH_2_), 29.0 (d, ^+^PCH_2_, ^1^*J*_CP_ = 53.1 Hz), 28.9 (s, 4-CH_2_), 28.0 (s, 3-CH_2_) 25.7 (d, 2-CH_2_). ^31^P{^1^H} NMR (D_2_O ) δ 24.0 (s). The purified ligand (319 mg, 370 μmol) was stirred with Gd_2_O_3_ (100 mg, 276 μmol) in water, excess Gd_2_O_3_ removed and the solution lyophilised to afford the pure complex as a colourless powder (378 mg, 97.2%). Purity > 95% by HPLC. MALDI-TOF–MS for [M]^+^: calculated *m/z* 903.284, observed *m/z* 903.306.

2,2′,2″-(10-(2-Oxo-14-(triphenylphosphonio)-6,9,12-trioxa-3-azatetradecyl)-1,4,7,10-tetraazacyclododecane-1,4,7-triyl)triacetatogadolinium(III) trifluoroacetate (**10**): The phosphonium salt (2.06 g, 3.55 mmol) was added to excess NaN_3_ (717 mg, 11.02 mmol) in DMF (30 mL) affording a light yellow oil azide (1.4 g, 73.9%). Hydrogenation of the azide (537 mg, 0.99 mmol) using freshly prepared Raney nickel (*ca.* 100 mg) in MeOH (50 mL) under a H_2_ atmosphere (10 atm) at 50 °C for 72 h yielded the corresponding amine (376 mg, 73.6%) as a slightly yellow oil. Slow addition of this amine (224 mg, 0.43 mmol) in DMF (5 mL) to a stirred solution of DOTA-^*t*^Bu_3_ (259 mg, 0.45 mmol), HATU (214 mg, 0.56 mmol), and NMM (210 μL, 193 mg, 1.91 mmol) in DMF (15 mL) yielded the crude protected ligand, which was subsequently deprotected with neat TFA (10 mL), dissolved in H_2_O (100 mL), washed, purified by means of reverse-phase HPLC, and lyophilised to afford the pure ligand as a colourless powder (115 mg, 28.4%). MALDI-TOF–MS for [M^+^·^*t*^Bu_3_]: calculated *m/z* 992.59, observed *m/z* 992.36. MALDI-TOF–MS for [M^+^]: calculated *m/z* 824.40, observed *m/z* 824.42.^1^H NMR (D_2_O) δ 7.72- 7.51 (m, 15H, Ar, *p*-PhP^+^, *o*-PhP^+^, *m*-PhP^+^), 3.81–3.12 [m, 40H: 3.72–3.55 (br s, 12H, OCH_2_), 3.51 (br s, 2H, NCH_2_C(O)), 3.44 (br s, 2H, CH_2_NHC(O)), 3.39 (br s, 2H, ^+^PCH_2_), 3.32 (br s, 6H, NCH_2_C(O)O), 3.18 (br s, 16H, NCH_2_CH_2_N)]. ^13^C NMR (D_2_O) δ 164.1, 135.1 (d, Ar, *p*-PhP^+^, ^4^*J*_CP_ = 2.0 Hz), 133.6 (d, Ar, *m*-PhP^+^, ^3^*J*_CP_ = 7.0 Hz), 130.3 (d, Ar, *o*-PhP^+^, ^2^*J*_CP_ = 13.8 Hz), 116.9 (m), 69.8 (s, OCH_2_), 69.2 (s, OCH_2_), 68.6 (s, OCH_2_), 58.0, 56.7, 50.8–49.3 (br m, NCH_2_CH_2_N), 41.6 (s, CH_2_NC(O)), 31.2 (d, ^+^PCH_2_, ^1^*J*_CP_ = 63.4 Hz). ^31^P{^1^H} NMR (D_2_O) δ 24.5 (s). *GP3* The purified ligand (110 mg, 117 μmol) was stirred with Gd_2_O_3_ (32 mg, 88 μmol) in water, excess Gd_2_O_3_ removed and the solution lyophilised to afford the pure complex as a colourless powder (128 mg, 96.8%). Purity > 95% by HPLC. MALDI-TOF–MS for [M]^+^: calculated *m/z* 979.300, observed *m/z* 979.272.

### Solution stability assays

Solution stability assays were performed using the method described by Barge et al.^32^. Initially an acetate buffer solution (50 mM) was prepared, a portion of which was adjusted to pH 5.8 using NaOH, while other portions were adjusted to pH 5.0, 4.0, or 3.0 using glacial acetic acid. Xylenol orange (5.81 mg) was dissolved in the pH 5.8 acetate buffer (48.4 mL) to give a 10 × concentrated stock solution. These solutions were treated as light sensitive, work was conducted in minimal light and solutions were covered in foil.

25 mM stock solutions of each GdCl_3_, **1** and **11** were prepared in the various buffer solutions. Two serial dilutions of each solution were freshly prepared, the first ranging from 25 mM to 19.5 μM, and the second ranging from 20 mM to 31.2 μM. To the wells of a 384 well plate were added 80 μL of the desired pH buffer and 10 μL of the selected serial dilution (in triplicate). The plates were agitated in a plate reader and allowed to equilibrate for 24 h. To each of the wells was added 10 μL of the 10 × concentrated xylenol orange stock solution in the absence of light. The plates were again agitated in a plate reader and allowed to equilibrate for 15 min at RT. The absorbance values were then determined at both 573 nm and 433 nm by means of a BMG LABTECH POLARstar Omega, UV Spectrometer, and the data plotted and processed using Graphpad Prism 7.

### Cell culture studies

The human glioblastoma multiforme (T98G) and human glial (SVG p12) cell lines were purchased from the ATCC and were maintained as monolayers in a minimum essential medium supplemented with foetal bovine serum (10% *v/v*), penicillin (100 units mL^−1^), streptomycin (100 μg mL^−1^) and L-glutamine (2.5 mM), within incubators at 37 °C in a humidified 5% CO_2_ atmosphere. Centrifugation was performed at 3000 rpm for 5 min. Earls MEM, FCS, PBS, Trypsin, L-glutamine, and the antibiotic solutions were purchased from ThermoFisher scientific.

### Cytotoxicity assays

The in vitro cytotoxicities of all Gd(III) complexes were assessed in both T98G and SVG p12 cell lines using the colourimetric 3-(4,5-dimethylthiazol-2-yl)-2,5-diphenyltetrazolium bromide (MTT) assay^47^. Briefly, cells were harvested with trypsin (0.1% *v/v*), the trypsin diluted with complete medium, and cell pellets isolated via centrifugation. Any remaining traces of trypsin were removed from the pellet via a suspension/centrifugation step. The pellets were then completely re-suspended, the number of cells mL^−1^ was determined using a hemocytometer (Weber), and the solution diluted such that 90 μL aliquots of cell suspension per well of a 96-well plate yielded 1 × 10^4^ cells per well. The cells were incubated for 24 h to allow them to adhere.

The cells were subsequently dosed with 10 μL per well of a sterile 10 × stock of the serial dilution (2 mM–62.5 μM) of Gd(III) complex (**1**–**11**) or the relevant vehicle (control). Each dilution/vehicle pair was repeated in triplicate within a plate and each plate was repeated in triplicate, for both cell lines. After 72 h incubation, MTT solution in phosphate-buffered saline (PBS; 30 μL, 0.17% *w/v*) was added to all wells in the absence of light and the incubation was continued for a further 4 h. The culture medium and excess MTT solution were carefully removed so as not to disrupt any MTT–formazan crystals formed during the incubation and were subsequently dissolved in 150 μL of DMSO.

Cell viability was determined by measuring the absorbance at 600 nm using a BMG LABTECH POLARstar Omega UV spectrometer. All readings were corrected for absorbance from the background control wells, and the level of MTT was expressed relative to the corresponding vehicle-treated controls (as % viability). Corresponding IC_50_ values for each of the compounds assessed were then determined as the dose required to give a 50% decrease in cell viability. IC_50_ values were reported with standard errors.

### Cell uptake studies

25 cm^3^ cell culture flasks were seeded with 3 mL of a cell suspension to yield 2 × 10^5^ cells per flask and were incubated for 72 h until ~ 80% confluent. Sterile 20 mM stock solutions of the Gd(III) complexes **1**–**10** were diluted with warm (37 °C) culture medium to yield a series dilution of final concentrations including 1000 μM, 500 μM, 250 μM, 125 μM and 62.5 μM. The non-dosed culture medium in each flask was removed and replaced with Gd-dosed media or a relevant vehicle (control), each concentration was repeated in triplicate for both cell lines along with the relevant control. The cells were incubated for a further 48 h after which they were harvested.

The culture medium was removed, and the monolayers washed with warm (37 °C) PBS (1 mL) prior to treatment with trypsin (0.1% *v/v*). The trypsin was diluted with medium, and cell pellets isolated by means of centrifugation before the supernatant was removed and the pellets re-suspended in warm PBS. Further centrifugation produced washed pellets, eliminating the possibility of residual Gd(III) complex remaining outside of the cells, which were subsequently re-suspended in warm PBS (1 mL). From this suspension, 100 μL was set aside for protein analysis and the remaining 900 μL was centrifuged to isolate the cell pellets. The supernatant was removed, and the cells were digested in concentrated HNO_3_ (110 μL, 69%) at 40 °C in a heating block for 24 h. 100 μL of the digest solution was diluted to 10 mL to result in a 1% HNO_3_ solution which was measured for Gd content by means of ICP-MS. ICP-MS was run on a Perkin Elmer ELAN 6100 Inductively Coupled Plasma Emission Mass Spectrometer (ICP-MS). Gd uptake is reported as μg Gd/mg protein ± standard error.

### Protein analysis

A Bio-Rad DC protein assay kit was used to determine protein concentrations^48^. A bovine serum albumin (BSA) protein standard curve was prepared each time the assay was performed, ranging from 1.0 to 0.1 mg mL^−1^ in PBS. Lysis of the 100 μL cell suspension was achieved using three snap freeze-heat cycles and thorough pipette mixing. The protein content of each solution was then analysed by pipetting 5 μL samples of the blanks, vehicle (control), and treated cell solutions into a 96-well plate, adding 25 μL of the alkaline copper tartrate solution and 200 μL of the Folin reagent solution included in the Bio-Rad kit in the absence of light. Each sample was prepared in triplicate. The plates were incubated at 37 °C for 15 min and the absorbance measured at 750 nm using a BMG LABTECH POLARstar Omega UV spectrometer. The protein concentration was determined by correcting all measurements with the background controls and comparing the absorbance with that of the BSA standard curve.

## Supplementary Information

Supplementary Information.
