# Supplementary material for: Synthesis and tumour cell uptake studies of gadolinium(III)–phosphonium complexes
Source: Sci Rep. 2021 Jan 12;11:598. doi: 10.1038/s41598-020-79893-9 (PMC7804430; doi:10.1038/s41598-020-79893-9)
Supplement: Supplementary file 1 — Supplementary Information. [file 41598_2020_79893_MOESM1_ESM.docx]

Supplementary Information

Synthesis and tumour cell uptake studies of gadolinium(III)-phosphonium complexes

Andrew J. Hall,^1^ Amy G. Robertson,^1^ Leila R. Hill ^1^ and Louis M. Rendina *^1,2^

^1^ School of Chemistry , The University of Sydney, Sydney, NSW 2006, Australia.

Email: [lou.rendina@sydney.edu.au](mailto:lou.rendina@sydney.edu.au)

^2^ The University of Sydney Nano Institute, Sydney, NSW 2006, Australia.

**Table S1** Cell uptake values for complexes **6**-**10** in SVG p12 and T98G cell lines at 62.5 μM.

|  | ng Gd/mg protein | |  |
| --- | --- | --- | --- |
| Complex | SVG p12 | T98G | T:N |
| **6** | 93.5 ± 8.1 | 158.8 ± 4.0 | 1.7 |
| **7** | 132.4 ± 5.6 | 231.0 ± 18.5 | 1.7 |
| **8** | 103.5 ± 23.6 | 237.5 ± 23.8 | 2.3 |
| **9** | 78.2 ± 8.6 | 175.6 ± 9.8 | 2.2 |
| **10** | 684.8 ± 63.8 | 1888.8 ± 203.5 | 2.8 |

*Where T:N is the tumour-to-normal cell ratio.*

**Table S2** Cell uptake values for complexes **1**-**10** in SVG p12 and T98G cell lines at 125 μM.

|  | ng Gd/mg protein | |  |
| --- | --- | --- | --- |
| Complex | SVG p12 | T98G | T:N |
| **1** | 133.9 ± 6.0 | 315.6 ± 30.5 | 2.4 |
| **2** | 444.2 ± 33.9 | 634.8 ± 27.3 | 1.4 |
| **3** | 462.3 ± 21.9 | 483.8 ± 16.5 | 1.0 |
| **4** | 412.5 ± 17.2 | 727.1 ± 6.5 | 1.8 |
| **5** | 351.2 ± 16.0 | 543.1 ± 30.8 | 1.5 |
| **6** | 694.5 ± 45.7 | 2534.5 ± 215.3 | 3.6 |
| **7** | 1810.2 ± 176.2 | 2741.3 ± 242.8 | 1.5 |
| **8** | 1435.1 ± 128.2 | 3156.4 ± 287.0 | 2.2 |
| **9** | 875.5 ± 57.9 | 2786.2 ± 200.9 | 3.2 |
| **10** | 1520.7 ± 86.3 | 2381.3 ± 389.8 | 1.6 |
|  |  |  |  |

*Where T:N is the tumour-to-normal cell ratio.*

**Table S3** Cell uptake values for complexes **1**-**10** in SVG p12 and T98G cell lines at 250 μM.

|  | ng Gd/mg protein | |  |
| --- | --- | --- | --- |
| Complex | SVG p12 | T98G | T:N |
| **1** | 484.4 ± 34.5 | 637.3 ± 30.7 | 1.3 |
| **2** | 582.1 ± 39.6 | 1093.2 ± 44.7 | 1.9 |
| **3** | 1008.0 ± 133.7 | 1038.8 ± 80.9 | 1.0 |
| **4** | 812.1 ± 56.7 | 1053.0 ± 43.2 | 1.3 |
| **5** | 678.0 ± 40.7 | 1010.4 ± 48.2 | 1.5 |
| **6** | 1826.7 ± 118.5 | 3047.2 ± 151.4 | 1.7 |
| **7** | 2145.4 ± 217.2 | 5349.1 ± 478.1 | 2.5 |
| **8** | 1713.4 ± 182.9 | 3512.0 ± 207.0 | 2.0 |
| **9** | 1131.8 ± 100.5 | 3201.2 ± 129.7 | 2.8 |
| **10** | 4658.5 ± 178.1 | 5407.5 ± 218.0 | 1.2 |

*Where T:N is the tumour-to-normal cell ratio.*

**Table S4** Cell uptake values for complexes **1**-**10** in SVG p12 and T98G cell lines at 500 μM.

|  | ng Gd/mg protein | |  |
| --- | --- | --- | --- |
| Complex | SVG p12 | T98G | T:N |
| **1** | 1067.5 ± 42.8 | 1146.5 ± 36.3 | 1.1 |
| **2** | 1860.2 ± 69.7 | 2485.7 ± 212.0 | 1.3 |
| **3** | 1997.5 ± 59.2 | 2146.5 ± 189.5 | 1.1 |
| **4** | 2321.3 ± 79.6 | 2852.7 ± 140.8 | 1.2 |
| **5** | 1941.9 ± 64.0 | 1715.0 ± 103.4 | 0.9 |
| **6** | 3670.8 ± 751.6 | 3426.2 ± 344.1 | 0.9 |
| **7** | 4366.7 ± 1017.3 | 3472.5 ± 1098.0 | 0.8 |
| **8** | 2600.6 ± 504.3 | 3257.1 ± 115.7 | 1.3 |
| **9** | 2176.9 ± 287.1 | 1922.7 ± 477.0 | 0.9 |
| **10** | 4576.0 ± 354.6 | 5229.9 ± 222.3 | 1.1 |
|  |  |  |  |

*Where T:N is the tumour-to-normal cell ratio.*

**Table S5** Cell uptake values for complexes **1**-**5** in SVG p12 and T98G cell lines at 1000 μM.

|  | ng Gd/mg protein | |  |
| --- | --- | --- | --- |
| Complex | SVG p12 | T98G | T:N |
| **1** | 1949.1 ± 232.9 | 2469.9 ± 306.7 | 1.3 |
| **2** | 2958.0 ± 237.7 | 3732.4 ± 337.9 | 1.3 |
| **3** | 2471.7 ± 89.3 | 3908.9 ± 859.5 | 1.6 |
| **4** | 3278.3 ± 375.5 | 3652.5 ± 82.5 | 1.1 |
| **5** | 2965.1 ± 201.6 | 3239.7 ± 128.7 | 1.1 |
|  |  |  |  |

*Where T:N is the tumour-to-normal cell ratio.*

**Table S6** Data from *T*_1_ inversion recovery studies for complex **1** in H_2_O at 298 K (9.4 T).

| [**1**] / mM | *T*_1_ / ms | 1/*T*_1_ / s^-1^ |
| --- | --- | --- |
| 0.00 | 355.783 | 2.811 |
| 0.25 | 178.618 | 5.599 |
| 0.49 | 118.532 | 8.437 |
| 0.74 | 105.543 | 9.475 |
| 0.98 | 90.862 | 11.006 |
|  |  |  |

*Performed on a 400 MHz Bruker 400 Avance NMR instrument (9.4 T). 10 μL D_2_O was added to 0.5 mL of the H_2_O in order to aid with the NMR deuterium lock.*

**Figure S1** Calculation of relaxivity of complex **1** in H_2_O giving *r*_1_ = 7.0 ­­± 1.1 mM^-1^ s^-1^ (9.4 T, 298 K) from the gradient of the line of best fit.
